# Abnormal cortical atrophy and functional connectivity are associated with depression in Parkinson’s disease

**DOI:** 10.3389/fnagi.2022.957997

**Published:** 2022-08-31

**Authors:** Weifang Yin, Anming Li, Baiyuan Yang, Chao Gao, Yanfei Hu, Zhenglong Luo, Yuxia Li, Yongyun Zhu, Chuanbin Zhou, Hui Ren, Shimei Li, Xinglong Yang

**Affiliations:** ^1^Department of Geriatric Neurology, First Affiliated Hospital, Kunming Medical University, Kunming, China; ^2^Department of Neurology, Chengdu Seventh People’s Hospital, Chengdu, China; ^3^Department of Radiology, First Affiliated Hospital, Kunming Medical University, Kunming, China; ^4^Department of Anesthesia, Kunming Xishan District People’s Hospital, Kunming, China; ^5^Yunnan Provincial Clinical Research Center for Neurological Diseases, Kunming, China; ^6^Yunnan Province Clinical Research Center for Geriatric Disease, Kunming, China

**Keywords:** Parkinson’s disease, depression, cortical atrophy, functional connectivity, neuroimaging biomarkers

## Abstract

**Objective:**

This study aimed to investigate the association of altered cortical thickness and functional connectivity (FC) with depression in Parkinson’s disease (PD).

**Materials and methods:**

A total of 26 non-depressed PD patients (PD-ND), 30 PD patients with minor depression (PD-MnD), 32 PD patients with major depression (PD-MDD), and 30 healthy controls (HC) were enrolled. Differences in cortical thickness among the four groups were assessed, and the results were used to analyze FC differences in regions of cortical atrophy. Binary logistic regression and receiver operating characteristic (ROC) curve analyses were also performed to identify clinical features and neuroimaging biomarkers that might help in the prediction of PD-MDD.

**Results:**

Patients with PD-MDD showed decreased cortical thickness compared to patients with PD-ND in the left superior temporal and right rostral middle frontal gyri (RMFG), as well as weak FC between the left superior temporal gyrus and right cerebellum posterior lobe and between right RMFG and right inferior frontal gyrus and insula. The combination of cortical thickness, FC, and basic clinical features showed strong potential for predicting PD-MDD based on the area under the ROC curve (0.927, 95% CI 0.854–0.999, *p* < 0.001).

**Conclusion:**

Patients with PD-MDD show extensive cortical atrophy and FC alterations, suggesting that cortical thickness and FC may be neuroimaging-based diagnostic biomarkers for PD-MDD.

## Introduction

Depression is prevalent among patients with Parkinson’s disease (PD), with approximately 10–90% of them reporting symptoms of depression and 17% being diagnosed with major depressive disorder (MDD) (Martinez-Martin et al., 2007; Reijnders et al., 2008; Brown et al., 2011; Schapira et al., 2017), a marker of prodromal PD and the malignant PD phenotype (Schapira et al., 2017). Depression in PD impairs mood and affection, and it is also frequently associated with other neuropsychiatric symptoms and complications later in life, impacting the quality of life and health outcomes (Chuquilin-Arista et al., 2020). Depression may occur at any stage in the course of PD, but depressive symptoms have been found to precede motor manifestations of PD in up to 50% of patients (Postuma et al., 2012; Schapira et al., 2017). In one study (Seritan et al., 2019), among PD patients whose depression predated motor symptoms, the mean age at onset of depressive disorders was 17.6 years earlier compared with the mean age at PD diagnosis. Despite the profound impact of depression on PD progression and manifestations, it is often under-recognized, and only 20–25% of depressed PD patients receive nursing or antidepressant therapies (Weintraub et al., 2003). Moreover, the pathophysiology of depression with underlying PD is poorly understood.

Neuroimaging studies have linked depression in PD to cortical atrophy in the prefrontal, limbic system, and cortical regions (Huang et al., 2016; Luo et al., 2016; Hanganu et al., 2017), as well as to increased precuneal thickness (Zanigni et al., 2017). Resting-state functional magnetic resonance imaging (RS-fMRI) of PD patients with depression revealed strong functional connectivity (FC) in the limbic and motor regions and weak FC between the cortical–limbic and cortical–cerebellar networks (Hu et al., 2015; Morgan et al., 2018; Liao et al., 2020). Abnormal FC between distinct neural circuits has also been observed even in PD patients with subclinical neuropsychiatric symptoms (Tinaz et al., 2021), and analysis of resting-state FC has shown potential for distinguishing between PD patients who are depressed or not (Lin et al., 2020).

While structural and functional MRI studies make clear that depression in PD is associated with abnormalities in brain structure, function, and networking (Prange et al., 2019; Zhang et al., 2019; Qiu et al., 2021), only a few studies have explored whether those abnormalities affect FC in the same population. In addition, most previous studies have focused on MDD, rather than on minor depression (MnD). MnD is a prevalent subclinical depressive condition characterized by a depressed mood or lack of interest along with 1–3 other depressive symptoms that persist for more than 2 weeks. MnD is more prevalent than MDD in the elderly (Polyakova et al., 2014), and it can considerably impair a patient’s quality of life, leading to functional disability or even increased risk of MDD or suicide attempts (Lyness et al., 2006; Pfeil et al., 2017). To our knowledge, only a few studies have investigated the association of MnD with cortical alterations, and the results have been conflicting. For instance, a previous study showed that the cortical thickness in patients with late-life MnD was lower than in healthy subjects (Kumar et al., 2014), whereas another study found no significant difference in the cortical thickness or gray matter volume between the two groups (Polyakova et al., 2020). Although the pathophysiology of MnD remains largely unexplored, its clinical similarity to MDD makes it a good model for examining early pathophysiological changes in depression. Therefore, we wanted to examine MnD in PD.

In the present study, we explored the underlying neuropathology of MnD and MDD in PD patients based on neuroanatomical and RS-fMRI analyses. The relationship between cortical thickness and depressive symptoms in PD was explored using surface-based morphometry (SBM), while FC in regions of cortical atrophy was analyzed using brain regions with different cortical thicknesses as seed points. Binary logistic regression and receiver operating characteristic curve (ROC) analyses were performed to identify clinical features and neuroimaging biomarkers that might help predict PD-MDD.

## Materials and methods

### Participants

A total of 26 non-depressed PD patients (PD-ND), 30 PD patients with MnD (PD-MnD), 32 PD patients with MDD (PD-MDD), and 30 healthy controls (HC) were recruited from the First Affiliated Hospital of Kunming Medical University between June 2020 and February 2022. The study was approved by the Ethical Committee of the First Affiliated Hospital of Kunming Medical University, and informed consent was provided by all participants.

### Inclusion and exclusion criteria

Patients were included if they (1) were right-handed, (2) met the Movement Disorder Society (MDS) Clinical Diagnostic Criteria for PD (Postuma et al., 2015), and (3) were diagnosed with MnD or MDD according to the 5th edition of the Diagnostic and Statistical Manual of Mental Disorders (Starkstein et al., 2011). We excluded (1) patients with PD or Parkinson’s superimposed syndrome caused by another disease, (2) patients with dementia, (3) patients diagnosed with psychiatric disorders, (4) patients who were currently receiving, or had ever received, antidepressants, (5) patients with a history of dementia, cerebrovascular disease, epilepsy, or other nervous system diseases, (6) patients who received intracranial surgical treatment, and (7) patients with contraindications to MRI. HCs were healthy individuals, matched to patients in age and sex, who had not been diagnosed with any neuropsychiatric disorder, such as depression or dementia.

### Clinical and neuropsychological evaluations

All clinical and neuropsychological evaluations were performed in a quiet and comfortable room during the off period. Data were collected from all subjects on age and sex, while data on education, course of the disease, medical history, age at onset, and levodopa equivalent daily dosage (LEDD) were collected from the PD patients. The severity of motor symptoms in PD patients was assessed using the MDS Unified PD Rating Scale part III (MDS-UPDRS-III) and the Hoehn and Yahr (H&Y) scale. The severity of depressive symptoms was evaluated using the 17-item Hamilton Rating Scale for Depression (HAMD-17), and anxiety was assessed using the Hamilton Anxiety Rating Scale (HAMA). Cognition function in PD patients was assessed using the Mini-mental State Examination (MMSE). The ratio of the mean UPDRS tremor scores (TD, 11 items) to the mean UPDRS postural instability/gait difficulty (PIGD) scores (5 items) was used to define the phenotypes of PD (Stebbins et al., 2013). If the resultant ratio is ≥1.15, then the patient is classified with TD. If the ratio is ≤0.90, then the patient is classified with PIGD. If the ratio is between 0.90 and 1.15, then the patient is classified as indeterminate (Stebbins et al., 2013).

### Magnetic resonance imaging data acquisition

All MRI data were collected on a 3T whole-body scanner (Discovery 750w, GE Healthcare, Fairfield, CT, United States) in the Department of Radiology at the First Affiliated Hospital of Kunming Medical University using standard head coils as transmitting and receiving coils. Before scanning, all participants were informed about the scanning requirements, and were then asked to lie down and relax without falling asleep or thinking. All subjects were asked to wear earplugs and keep their head still to minimize head movement. Three-dimensional T1-weighted magnetization-prepared rapid gradient echo sagittal images were acquired using the following parameters: 156 slices; slice thickness, 1 mm; no gap; voxel size, 1 × 1 × 1 mm; repetition time, 1,900 ms; echo time, 2.0 ms; inversion time, 450 ms; flip angle, 12°; field of view, 256 mm; matrix, 256 × 256. RS-fMRI images were obtained with echo-planar imaging using the following parameters: 36 slices; slice thickness, 3 mm; no gap; voxel size, 3.5 × 3.5 × 4 mm; volume, 240; repetition time, 2,000 ms; echo time, 30 ms; flip angle, 90°; field of view, 224 mm; matrix, 64 × 64.

### Magnetic resonance imaging data processing

#### T1 imaging

The MRI scans were screened to exclude clinically significant abnormalities and poor-quality images. To analyze cortical thickness, T1-weighted images were processed with FreeSurfer version 6.0.0^1^ in the Ubuntu Linux system and the SBM method (Fischl, 2012). Reformatted MRI images were then preprocessed using the standard recon-all pipeline. Magnetic inhomogeneity was corrected, the skull was stripped, and images were segmented into the gray and white matter. Then cortical tissue boundaries were reconstructed and transformed into a subject-specific surface mesh, resulting in a triangular cortical mesh for gray and matter surfaces consisting of approximately 150,000 vertices/hemisphere. Next, three-dimensional inflated and spherical white matter models were created by inflating the cortical sulci for spatial normalization and further statistical analysis. The data obtained for each subject were visually inspected to ensure accuracy of registration, skull stripping, segmentation, and cortical surface reconstruction. The full width at half-maximum (FWHM) was set to 15 mm for statistical analysis (Dale et al., 1999).

#### Resting-state functional magnetic resonance imaging pre-processing

The RS-fMRI images were analyzed using the toolbox for Data Processing and Analysis of Brain Imaging (version 4.5)^2^ based on the Statistical Parametric Mapping software (SPM12^3^) and the MATLAB platform version 2013b (The MathWorks, Natick, MA, United States^4^) (Yan et al., 2016). The first 10 time points were removed to avoid artifacts due to scanner calibration and subjects’ adaptation to the scanning environment. The remaining images were then corrected for slice timing using the middle slice as a reference and realigned to remove head motion. To further minimize the influence of head motion on FC analysis, we removed images with a head displacement greater than 2 mm or angle rotation > 2° in the *x, y*, or *z* directions. Ten subjects were excluded due to head motion.

The T1-weighted anatomical images were co-registered to the mean functional images using rigid-body transformation. The structural images were then segmented into gray matter, white matter, and cerebrospinal fluid using the DARTEL template. Data were also normalized to the Montreal Neurological Institute (MNI) space using the DARTEL template to obtain anatomical accuracy across participants, and the normalized RS-fMRI data were resampled to voxels of 3 × 3 × 3 mm^3^ with spatial smoothing based on a Gaussian kernel with isotropic FWHM of 8 mm. Next, low-frequency drifts and physiological high-frequency noise were removed using linear detrending and temporal bandpass filtering (0.01–0.08 Hz). Regression was performed on the time course of nuisance covariates, including white matter and cerebrospinal fluid signals, as well as Friston-24 head motion parameters, including six head motion parameters, their historical effects, and 12 corresponding squared items (Gallea et al., 2021).

#### Functional connectivity analysis

To compare FC across the groups of subjects, we compared them in brain regions with altered cortical thickness, which were defined as regions of interest (ROIs). These ROIs were used as seed regions for voxel-wise FC analysis. The Pearson correlation coefficient between the average time series within the ROIs and other brain areas was calculated, and the resulting connectivity maps were transformed into Z maps using Fisher’s r-to-z transformation.

### Statistical analysis

#### Statistical analysis of clinical data

Clinical data were statistically analyzed using SPSS 23.0 (IBM, Chicago, IL, United States). Data showing normal distribution were reported as mean ± standard deviation (SD). Differences between two groups were assessed for significance using an independent-samples *t*-test, while differences among three or more groups were assessed using analysis of variance. Skewed data were reported as median (interquartile range), and differences between two groups were assessed using the Mann–Whitney *U* test, while differences among three or more groups were assessed using the Kruskal–Wallis *H* test. Differences in count data were assessed using the chi-squared test. Differences associated with *P* < 0.05 were considered statistically significant.

#### Statistical analysis of T1-magnetic resonance imaging data

The T1-MRI data were statistically analyzed using a vertex-by-vertex general linear model (GLM) in FreeSurfer, in which cortical thickness was the dependent factor, the diagnosis was the independent factor, and age, sex, and education were nuisance variables. All results were corrected for multiple hypothesis testing using pre-cached cluster-wise Monte Carlo simulation. Statistical significance was set to a cluster-forming *P* < 0.001 and cluster-wise probability (CWP) < 0.05 after correction with 10,000 permutations. A Bonferroni correction method was used to further correct the error rate for the analysis of GLM. First, we analyzed the brain regions across the PD-ND, PD-MnD, PD-MDD, and HC groups, and then we evaluated the differences among the three PD patient groups. Correlations between HAMD score and cortical thickness were also assessed. Then, clusters with significant interactions on cortical thickness were defined as regions of interest (ROIs) for FC analysis, and the mean cortical thickness within each cluster was extracted for regression and ROC curve analysis.

#### Statistical analysis of resting-state functional magnetic resonance imaging data

Functional connectivity was analyzed statistically using Z maps that showed a normal distribution within SPM12 in MATLAB, with age, sex, and education as covariables. FC values from different seed points to the whole brain in different groups were compared using a two-sample *t*-test. All tests were two-tailed, and all statistical results were corrected using a Gaussian Random Field. Data associated with voxel-level *P* < 0.001 and cluster-level *P* < 0.05 were considered statistically significant. Mean FC values differing significantly between the PD-MDD and PD-ND groups were also analyzed in terms of correlations, binary logistic regression, and receiver operating characteristic (ROC) curves. Cutoff values for generating ROC curves were selected using the Youden index and the minimum distance from the coordinate (0,1).

## Results

### Clinicodemographic characteristics

No significant differences were observed in age or sex among all four groups, or in the age at onset or LEDD among the three groups of PD patients (Table 1). However, PD-MDD patients had higher UPDRS III, H&Y, HAMD, and HAMA scores than patients with PD-ND and PD-MnD. Besides, analysis of PD subtypes showed that the proportion of PIGD subtypes in PD-MDD was higher than that in PD-ND patients.

**TABLE 1:** Clinicodemographic characteristics of study subjects.

Characteristic	HC	PD-ND	PD-MnD	PD-MDD	*P*
Participants	30	26	30	32	
Age, years	62.9 ± 10.8	61.8 ± 12.7	61.2 ± 9.8	65.4 ± 10.3	0.435
Male	13 (43.3%)	19 (73.1%)	18 (60%)	18 (56.3%)	0.161
Age of onset	NA	59.59 ± 12.74	57.9 ± 9.81	60.79 ± 10.57	0.586
Disease duration	NA	1.75 (0.5, 2.0)^a*,b**^	3 (1,5)^a*^	4 (1.75, 5.5)^b**^	0.01
LEDD (mg/day)	NA	337.5 (150, 375)	268.75 (187.5, 375)	375 (277.5, 568.75)	0.05
UPDRS III	NA	17 (14, 26)^b**^	22.5 (15, 32)^c**^	41 (24.5, 43.5)^b**,c**^	<0.001
H&Y stage	NA	1 (1, 2)^b**^	2 (1, 2)^c**^	3 (2, 3)^b**,c**^	<0.001
HAMD	NA	4.57 ± 3.43^a**,b**^	12.23 ± 4.07^a**,c**^	23.75 ± 7.77^b**,c**^	<0.001
HAMA	NA	8.38 ± 7.29^a*,b**^	16.06 ± 9.30^a*,c**^	24.87 ± 11.93^b**,c**^	<0.001
MMSE	NA	28 (24, 29)	26 (22, 28)	25 (24, 26)	0.072
Subtype of PD (TD)	NA	13 (50%)^b*^	10 (33.3%)	6 (18.8%)^b*^	0.036
Subtype of PD (PIGD)	NA	12 (46.2%)^b*^	19 (63.3%)	20 (62.5%)^b*^	

Values are n, n (%), mean ± SD, or median (interquartile range), unless otherwise noted.

*P < 0.05, **P < 0.01.

^a^PD-ND vs. PD-MnD, ^b^PD-ND vs. PD-MDD, ^c^PD-MnD vs. PD-MDD.

HAMA, Hamilton Anxiety Rating Scale; HAMD, Hamilton Rating Scale for Depression; HC, healthy controls; LEDD, levodopa equivalent daily dosage; MMSE, Mini-mental State Examination; PD, Parkinson’s disease; PD-ND, non-depressed PD patients; PD-MnD, PD patients with minor depression; PD-MDD, PD patients with major depressive disorder; UPDRS III, Unified PD Rating Scale part III; TD, tremor dominant; PIGD, postural instability/gait difficulty.

### Cortical thickness and functional connectivity

Comparison of cortical thickness in the four groups revealed significant differences between patients with PD-MDD and those with PD-MnD or PD-ND, as well as between the PD-MDD group and healthy individuals. In contrast, no significant difference was observed between HC and the PD-ND or PD-MnD groups, or between the PD-ND and PD-MnD groups (Table 2).

**TABLE 2:** Intergroup comparisons of cortical thickness in different brain regions.

Brain region	Cluster size	MNI coordinates			Vertices	*P*
		
		X	Y	Z		
**Cortex thinner in PD-MDD than in HC**						
Left fusiform gyrus	4169.65	−34.7	−33.7	−25.2	7379	<0.0001
Left pars opercularis	855.30	−41.3	26.6	13.6	1336	0.0002
Right fusiform gyrus	2202.52	33.1	−45.9	−14.2	4054	<0.0001
Right temporal pole	1342.82	30.5	2.4	−29.6	2334	0.0001
Right middle temporal	1329.78	53.2	−12.6	−21.0	2353	<0.0001
Right lateral orbitofrontal	1094.30	34.2	33.6	−8.2	2118	0.0002
Right lateral occipital	830.12	33.7	−88.9	−6.1	1055	<0.0001
Right post-central	778.46	46.9	−18.1	55.7	1556	<0.0001
**Cortex thinner in PD-MDD than in PD-MnD**						
Left fusiform gyrus	2781.41	−32.4	−45.5	−15.0	4777	<0.0001
Right fusiform gyrus	1232.61	32.0	−49.5	−17.1	2197	<0.0001
**Cortex thinner in PD-MDD than in PD-ND**						
Left fusiform gyrus	4298.42	−30.7	−57.1	−12.1	6354	<0.0001
Left lateral occipital cortex	1101.50	−28.3	−90.9	−3.3	1460	0.0005
Left superior temporal gyrus	726.48	−41.2	−17.7	−10.5	1874	0.0001
Left lateral orbitofrontal cortex	713.69	−29.6	28.1	−16.6	1367	0.0007
Right fusiform gyrus	3785.34	33.3	−51.2	−6.6	5349	0.0003
Right middle temporal gyrus	1614.58	50.9	−13.6	−20.1	2641	<0.0001
Right RMFG	1042.61	37.5	33.1	10.5	1735	0.0002
**HAMD correlation**						
Left fusiform gyrus	1954.98	−31.3	−68.7	−14.9	2507	0.0001
Left lingual gyrus	907.86	−9.1	−73.4	−2.6	1499	0.0002
Right lateral occipital cortex	1367.21	21.6	−93.8	−9.5	1691	<0.0001

HAMD, Hamilton Rating Scale for Depression; HC, healthy controls; MNI, Montreal Neurological Institute; PD, Parkinson’s disease; PD-ND, non-depressed PD patients; PD-MnD; PD patients with minor depression; PD-MDD, PD patients with major depressive disorder; RMFG, rostral middle frontal gyrus.

The brain regions with altered cortical thickness were used as ROIs to analyze changes in FC. A total of 17 ROIs were identified, of which seven showed FC alterations. Intergroup comparison revealed significant differences between HC and patients with PD-MDD or PD-ND, whereas no significant FC differences were observed between the structurally altered brain regions of PD-MDD and PD-MnD (Table 3).

**TABLE 3:** Intergroup comparison of functional connectivity in different brain regions.

ROI [MNI coordinates (X, Y, Z)]	Brain regions	MNI coordinates			Vertices	*T* value
		
		X	Y	Z		
**FC difference when PD-MDD minus HC**						
Left pars opercularis (−41.3, 26.6, 13.6)	Right midbrain limbic lobe	3	−30	−15	327	−4.0879
Right fusiform gyrus (33.1, −45.9, −14.2)	Left parietal lobe	−45	−54	42	476	−4.4103
	Limbic lobe cingulate gyrus	−6	−24	42	360	−4.2659
Right middle temporal gyrus (53.2, −12.6, −21.0)	Bilateral occipital lobe Right cerebrum	18	−102	−9	1328	−5.4053
Right lateral orbitofrontal cortex (34.2, 33.6, −8.2)	Right cerebellum	24	−51	−54	528	4.1848
Right lateral occipital cortex (33.7, −88.9, −6.1)	Right temporal lobe	54	−27	−9	463	−4.5896
**FC difference when PD-MDD minus PD-ND**						
Left superior temporal gyrus (−41.2, −17.7, −10.5)	Right cerebellum posterior lobe	33	−60	−21	447	−4.5084
Right RMFG (37.5, 33.1, 10.5)	Right inferior frontal gyrus Right insula	63	12	0	707	−4.502

HC, healthy controls; MNI, Montreal Neurological Institute; PD, Parkinson’s disease; PD-ND, non-depressed PD patients; PD-MnD, PD patients with minor depression; PD-MDD, PD patients with major depressive disorder; RMFG, rostral middle frontal gyrus; ROI, region of interest.

**FIGURE 1:**
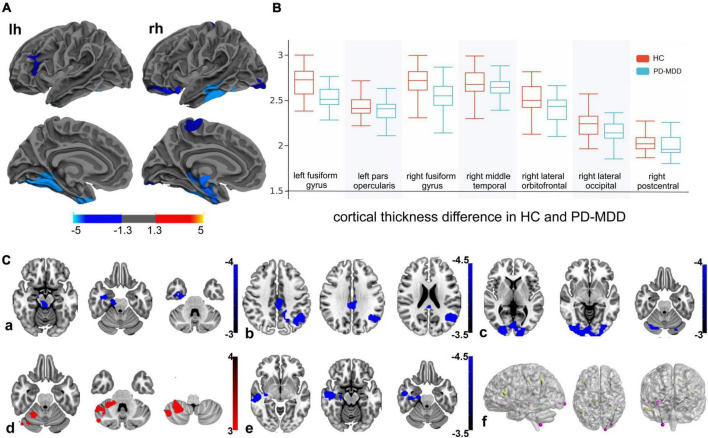
**(A)** Cortical thickness of the left hemisphere (lh) and right hemisphere (rh) in Parkinson’s disease patients with major depression (PD-MDD) and healthy controls (HC). The color bar indicates the –log_10_ value of *P* (red, higher cortical thickness; blue, lower cortical thickness). **(B)** Cortical thickness values of different clusters between PD-MDD and HC. **(C)** Differences in functional connectivity between the following regions of interest and the entire brain in PD-MDD and HC: (a) left pars opercularis, (b) right fusiform gyrus, (c) right middle temporal gyrus, (d) right lateral orbitofrontal cortex, (e) right lateral occipital cortex, and (f) regions showing significant differences in functional connectivity between the groups. The color bar represents the *T* value (red and blue indicate stronger or weaker functional connectivity, respectively).

Patients with PD-MDD showed decreased cortical thickness compared to HC in the bilateral fusiform gyrus, left pars opercularis, right temporal pole, right middle temporal gyrus, right lateral orbitofrontal cortex, right lateral occipital cortex, and right post-central gyrus (Figure 1). The PD-MDD group also showed weaker FC between (1) the left pars opercularis and right midbrain and the limbic lobe; (2) the right middle temporal gyrus and the bilateral occipital lobe and right cerebrum; (3) the right lateral orbitofrontal cortex and the right cerebellum; (4) the right lateral occipital cortex and the right temporal lobe; and (5) the right fusiform gyrus and the left parietal lobe, limbic lobe, and cingulate gyrus (Figure 1).

**FIGURE 2:**
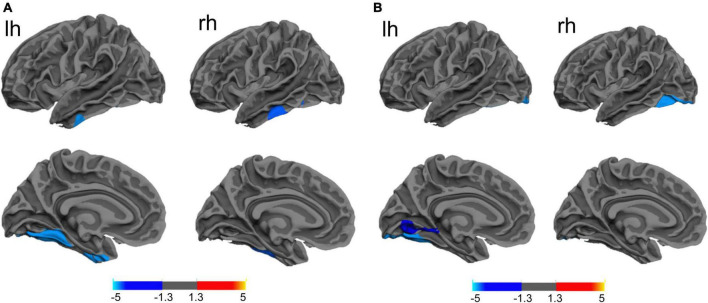
**(A)** The difference between Parkinson’s disease patients with major (PD-MDD) or minor depression (PD-MnD) in cortical thickness of the left hemisphere (lh) or right hemisphere (rh). **(B)** Correlation of cortical thickness of the left hemisphere (lh) and right hemisphere (rh) with the Hamilton Depression Rating Scale score in Parkinson’s disease patients. The color bar indicates the –log_10_ value of *P* (red, higher cortical thickness or positive correlation; blue, lower cortical thickness or negative correlation).

Patients with PD-MDD also showed decreased cortical thickness in the bilateral fusiform gyrus compared to the PD-MnD group (Figure 2), but no significant FC differences were identified between the two groups for any ROI.

**FIGURE 3:**
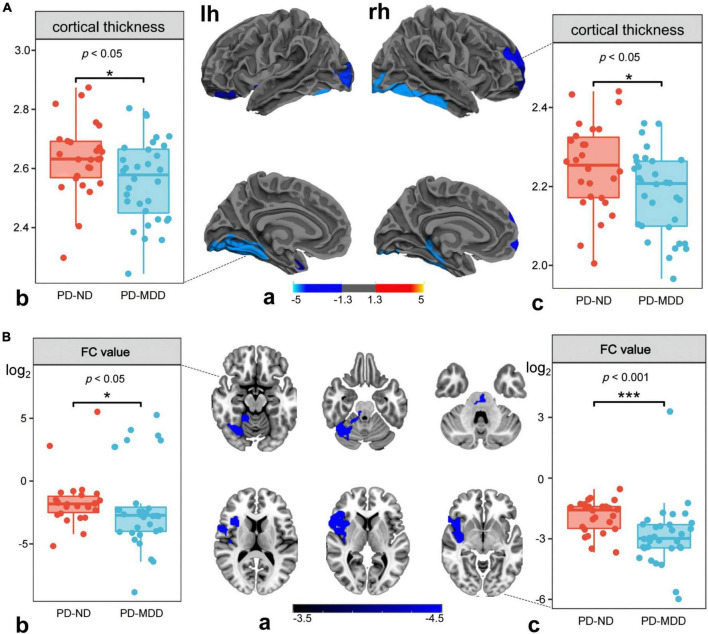
**(A)** (a) Differences between Parkinson’s disease patients with major (PD-MDD) or no depression (PD-ND) in cortical thickness of the left hemisphere (lh) or right hemisphere (rh). The color bar indicates the –log10 value of *P* (red, higher cortical thickness; blue, lower cortical thickness); the cortical thickness in (b) left superior temporal gyrus and (c) right rostral middle frontal gyrus. **(B)** (a) Differences between PD-MDD and PD-ND in functional connectivity (FC) between the entire brain and the regions of cortical atrophy and (b) the FC value between left superior temporal gyrus and right cerebellum posterior lobe, (b) the FC value between rostral middle frontal gyrus to right inferior frontal gyrus and right insula. The color bar represents the *T* value.

Similarly, a lower cortical thickness was observed in the PD-MDD group compared to patients with PD-ND in the bilateral fusiform gyrus, left lateral occipital cortex, left superior temporal gyrus, left lateral orbitofrontal cortex, right middle temporal gyrus, and right rostral middle frontal gyrus (RMFG) (Figure 3). In addition, the PD-MDD group showed weaker FC than the PD-ND group between the left superior temporal gyrus and the right cerebellum posterior lobe as well as between the right RMFG and the right inferior frontal gyrus and insula (Figure 3).

### Correlation of cortical thickness and functional connectivity with Hamilton Rating Scale for Depression score

The HAMD score negatively correlated with cortical thickness in the left fusiform gyrus, left lingual gyrus, and right lateral occipital lobe (Table 2 and Figure 2). In addition, the FC value between the left superior temporal gyrus (seed 1) and the right cerebellum posterior lobe, as well as between the right RMFG (seed 2) and the right inferior frontal lobe and insula negatively correlated with the HAMD score in PD-MDD patients (*r* = −0.426, *P* = 0.001) and PD-ND patients (*r* = 0.516, *P* < 0.001).

### Binary logistic regression and receiver operating characteristic curve analyses

We performed binary logistic regression and ROC curve analysis to distinguish patients with PD-MDD from patients with PD-ND. First, we extracted the cortical thickness of the left superior temporal gyrus (seed 1) and the right RMFG (seed 2), as well as the FC values between seed 1 and the right cerebellum posterior lobe and between seed 2 and the right inferior frontal gyrus and insula. Then, we converted the FC values to a logarithmic scale (log_2_) and used them along with basic clinical features of both patient groups to perform binary logistic regression. The results identified the UPDRS III score and the FC value between seed 2 and the right inferior frontal gyrus and insula as independent risk factors of PD-MDD (Table 4).

**TABLE 4:** Factors associated with Parkinson’s disease with major depression.

Factor	B	Exp (B)	95% CI	*P*
Age	−0.041	0.960	0.876–1.051	0.372
Disease duration	−0.146	0.864	0.650–1.151	0.318
UPDRS III score	0.101	1.007	1.010–1.213	0.030
H&Y stage	0.669	1.953	0.767–4.972	0.160
Subtype of PD (TD)	1.719	5.579	0.286–108.998	0.257
Subtype of PD (PIGD)	0.498	1.645	0.242–11.175	0.067
Cortical thickness of seed 1	−3.959	0.019	0.000–40.459	0.311
Cortical thickness of seed 2	−8.649	0.000	0.000–1.850	0.067
FC between seed 1 and right cerebellum posterior lobe	0.184	1.202	0.863–1.674	0.277
FC between seed 2 and right inferior frontal gyrus and insula	0.954	2.596	1.362–4.949	0.004

CI, confidence interval; FC, functional connectivity; H&Y, Hoehn and Yahr scale; UPDRS III, Unified PD Rating Scale part III; TD, tremor dominant; PIGD, postural instability/gait difficulty.

**FIGURE 4:**
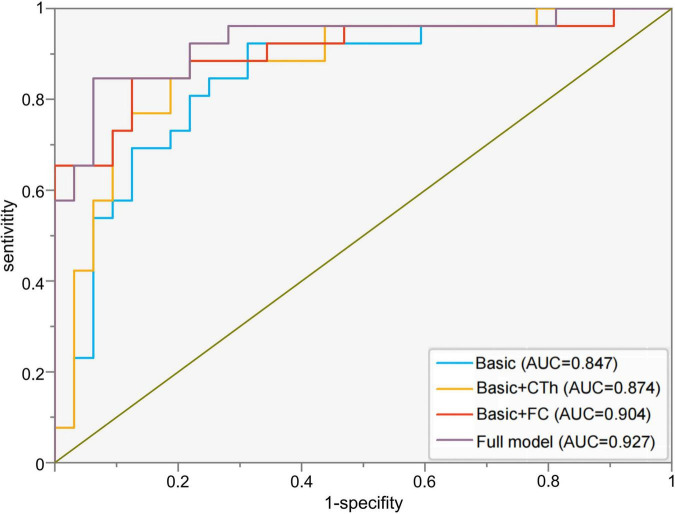
Receiver operating characteristic curves to assess the ability of different models to predict major depression in Parkinson’s disease. Basic model: clinical features (age, disease duration, UPDRS III score, H&Y stage, and subtype of PD); Basic + CTh model: clinical features and cortical thickness of left superior temporal gyrus (seed 1) and right rostral middle frontal gyrus (seed 2); Basic + FC: clinical features and FC values between seed 1 and the right cerebellum posterior lobe as well as between seed 2 and the right inferior frontal gyrus and insula; Full model: clinical features, CTh, and FC value. AUC, area under the receiver operating characteristic curve; CTh, cortical thickness; FC, functional connectivity.

A ROC curve analysis was also performed to establish an accurate diagnostic model for PD-MDD. The prediction accuracy increased gradually from the basic model (clinical features, AUC = 0.847, 95% CI 0.744–0.950, *p* < 0.001) to basic characteristics combined with the cortical thickness of two seeds (AUC = 0.874, 95% CI 0.779–0.969, *p* < 0.001). The prediction accuracy further increased with models using basic characteristics combined with FC values (AUC = 0.904, 95% CI 0.819–0.989, *p* < 0.001), and the highest specificity and sensitivity were achieved with the full model which combined basic clinical features (age, disease duration, UPDRS III score, H&Y stage, and subtype of PD) with the following neuroimaging biomarkers: the cortical thickness of the two seeds and the FC values between seed 1 and the right cerebellum posterior lobe as well as between seed 2 and the right inferior frontal gyrus and insula (Table 5 and Figure 4).

**TABLE 5:** Accuracy of different models for predicting Parkinson’s disease with major depression.

Model	AUC (95%CI)	Sensitivity	Specificity	*P*
Basic Age Disease duration UPDRS III score H&Y stage Subtype of PD	0.847 (0.744–0.950)	68.8%	61.1%	<0.001
Basic+ Cortical thickness of seed 1 Cortical thickness of seed 2	0.874 (0.779–0.969)	78.1%	66.6%	<0.001
Basic+ FC between seed 1 and right cerebellum posterior lobe FC between seed 2 and right inferior frontal gyrus and insula	0.904 (0.819–0.989)	87.5%	72.1%	<0.001
Full model*	0.927 (0.854–0.999)	93.8%	78.4%	<0.001

AUC, area under the receiver operating characteristic curve; CI, confidence interval; FC, functional connectivity; H&Y, Hoehn and Yahr scale; UPDRS III, Unified PD Rating Scale part III.

*Full model, combining basic clinical features (age, disease duration, UPDRS III score, H&Y stage, and subtype of PD) with the following neuroimaging biomarkers: cortical thickness of the two seeds and the FC values between seed 1 and the right cerebellum posterior lobe as well as between seed 2 and the right inferior frontal gyrus and insula.

## Discussion

The present study systematically investigated the structural and functional changes in the brain of patients with PD-MDD and PD-MnD. Compared to non-depressed patients, PD patients with major depression showed decreased cortical thickness in the left superior temporal gyrus and right RMFG, as well as weak FC between the left superior temporal gyrus and right cerebellum posterior lobe and between right RMFG and right inferior frontal gyrus and insula, suggesting that these factors may be potential neuroimaging biomarkers for PD-MDD. In addition, PD-MDD showed extensive cortical atrophy and functional changes when compared with PD-ND and HC. Besides, the PD-MnD patients also showed cortical atrophy in part of the brain regions when compared with PD-MDD.

The RMFG belongs to the dorsolateral prefrontal cortex (DLPFC), which is part of the prefrontal cortex that plays a key role in depression as a top-down emotion regulator (Phillips et al., 2003). Studies on depressive symptoms and cerebral cortex thickness have shown that the orbital, inferior, and superior frontal gyri are thinner in PD patients than in healthy individuals (Huang et al., 2016; Hanganu et al., 2017). In addition, one of these studies found PD patients with depression to have a thinner motor cortex and middle frontal gyrus than non-depressed PD patients, but thicker orbitofrontal and insular cortices (Huang et al., 2016). Another study found that the prefrontal cortex was thinner in drug-naive PD patients with depression compared to non-depressed PD patients (Luo et al., 2016). These results suggest that the cortical thickness in the frontal and prefrontal cortices is significantly associated with depression in PD. However, the prefrontal cortex and DLPFC are not adequately defined, and the disease mechanism has not been thoroughly investigated. We expect that the present study will help elucidate a small region within the DLPFC, which might play a crucial role in PD-MDD.

The RMFG is also located in the control network of the brain and is involved in the frontal-limbic modulation of cognitive control and affective processing, while contributing to the regulation of emotional availability and the recognition of emotions (Leon et al., 2021; Song et al., 2021). In addition, the RMFG is critical for higher-order executive functions, such as attention, working memory, planning, executive cognition, and emotion regulation (Koenigs and Grafman, 2009), and its thickness has been directly associated with depression (Michalski et al., 2017). The insula is also related to various emotional and cognitive functions in the human brain, and its dysfunction has been associated with depression in PD patients (Christopher et al., 2014; Huang et al., 2020; Jonkman et al., 2021). Another study correlated abnormal insular-frontal hypoconnectivity with persistent depressive symptoms or antidepressant effects (Morgan et al., 2018), and Lewy body pathology can extend to the anterior insular cingulate region and intermediate cortex, supporting the idea that insular dysfunction is an important cause of depressed PD (Braak et al., 2003). Consistent with these results, we found that patients with PD-MDD have reduced FC from the right RMFG to the right inferior frontal gyrus and insula, suggesting that the RMFG is significantly correlated with PD-MDD and that the corresponding FC value may be a potential neuroimaging biomarker for PD-MDD.

In this study, PD-MDD patients showed decreased cortical thickness in the left superior temporal gyrus as well as weak FC between the left superior temporal gyrus and the right cerebellum posterior lobe. The temporal cortex is involved in emotional face recognition and processing, and its altered cortical thickness and function have been associated with depression in PD (Liang et al., 2016; Hanganu et al., 2017; Morgan et al., 2018). According to Beck’s cognitive model of depression (Beck, 2008), depression is characterized by rigid, valence-specific biases in the processing of affective information, which create preferential processing of negative information and decreased engagement with positive information, thus affecting several processing domains, including attention, memory, interpretation, and implicit associations (Kalia and Lang, 2015; Price and Duman, 2020). The cerebellum is also involved in sensorimotor and vestibular control, as well as in cognition, emotion, and autonomic function (Schmahmann, 2019). Cerebellar posterior lobe lesions cause the cerebellar cognitive affective syndrome, which in turn leads to deficits in executive function, visual-spatial processing, linguistic skills, and regulation of effects (Schmahmann, 2019). Previous studies have associated depression in PD with cerebellum dysfunction and increased amplitudes of low-frequency fluctuations, indicating that depressed PD patients have enhanced cerebellum neural activity and FC between the frontal lobe and the cerebellum (Liang et al., 2016; Wei et al., 2017; Morgan et al., 2018; Lin et al., 2020). Thus, we conclude that the FC between the left superior temporal gyrus and the cerebellum plays a key role in the development of PD with depression.

Cortical atrophy and abnormal FC were also observed in the bilateral fusiform, left lateral occipital, left orbitofrontal, and right middle temporal gyri of our PD-MDD patients compared to patients with PD-ND and HC. The orbitofrontal cortex is crucial for emotional processing, and its decreased thickness has been associated with depression in PD (Luo et al., 2016; Hanganu et al., 2017). Previous studies reported abnormal functional activity and metabolism in the orbitofrontal cortex, as well as decreased FC between the orbitofrontal and fusiform gyri in depressed PD patients (Luo et al., 2014; Hu et al., 2015; Prange et al., 2019). Emotional face-specific clusters were also identified in regions involved in face processing, such as the anterior fusiform and middle temporal gyri, while emotional perception has been associated with the lateral occipital cortex (Sabatinelli et al., 2011).

At the neurocognitive level, depression is considered a disorder of impaired cognitive flexibility and prefrontal inhibition that leads to negative biases in cognition, such as rigidly held negative beliefs (Kashdan and Rottenberg, 2010; Sabatinelli et al., 2011). As Beck’s cognitive model of depression stipulates, depressed patients bias their attention to sad stimuli, triggering a feedback loop in the cognitive system, which in turn induces and sustains depression (Beck, 2008; Disner et al., 2011). Since PD-MDD patients in our study showed extensive cortical atrophy, we hypothesize that the cognitive model of depression may also apply to depression in PD. Nevertheless, a cognitive model of depression in PD may need to be more complex and involve more brain regions, given that PD is a heterogeneous disease with complex symptoms that interact with depression and lead to abnormal emotion recognition, processing, and integration along with prolonged depressive mood.

Compared to the PD-MDD group, patients with PD-MnD showed higher cortical thickness in the bilateral fusiform gyrus in our study. To the best of our knowledge, this is the first MRI study to investigate MnD in PD patients. Although the pathophysiology of MnD remains largely unexplored, it has been shown that the cortical thickness is reduced in similar brain regions as in patients with MDD (Kumar et al., 2014; Polyakova et al., 2020). Consistent with these results, we found that patients with PD-MnD and PD-MDD had comparable lesions in certain brain regions, implying that PD-MnD has similar neurobiological substrates to PD-MDD that may be involved in the pathophysiology of mood disorders. Our finding of a negative correlation between HAMD and cortical thickness in the left fusiform gyrus also suggests that cortical thickness is associated with the progression of depressive symptoms in PD.

Furthermore, our ROC analysis showed that the prediction accuracy of a diagnostic model for PD-MDD increased when the basic clinical characteristics were combined with cortical thickness and FC. Although several studies have explored the structural and functional alterations in depression and PD, their results are conflicting. Our findings lead us to suggest that cortical thickness and FC may be more accurate neuroimaging biomarkers for predicting and elucidating the mechanism of PD with depression, while cortical thickness may be a more suitable structural biomarker than gray matter volume (Polyakova et al., 2020).

Our study has some limitations, including the fact that it had a cross-sectional design with a relatively small sample. The cross-sectional design meant that we could not explore a causal relationship between structural and functional changes. We did not take into account the use of medication, which can be an important confounder, although the three groups of patients in our study did not differ significantly in doses of dopaminergic drugs. Moreover, depression has a strong correlation with cognitive disturbances in PD, although we carefully excluded patients with dementia, we cannot remove the impact of mild cognitive impairment. Maybe a detailed cognitive assessment would have provided more insight into the neural substrate of depression and the complex interplay of PD depression and cognition. Furthermore, we could not explore whether PD shares a common neurobiological substrate with primary depression, as we did not recruit individuals with primary depression. Therefore, studies with larger, clinically homogeneous samples are needed to validate and extend our findings.

Despite these limitations, our study provides strong evidence that changes in brain structure and function are strongly associated with an increased risk of major depression in PD. Our work also identifies cortical thickness and FC as promising neuroimaging biomarkers for predicting PD-MDD.

## Data availability statement

The raw data supporting the conclusions of this article will be made available by the authors, without undue reservation.

## Ethics statement

The studies involving human participants were reviewed and approved by the First Affiliated Hospital of Kunming Medical University. The patients/participants provided their written informed consent to participate in this study.

## Author contributions

WY, AL, SL, and XY: design of the work. WY, AL, BY, CG, and YH: implementation of the project and data acquisition and analysis. WY, AL, ZL, YL, YZ, CZ, CG, and YH: data collection. SL, XY, and HR: critical review and revision of the manuscript. All authors have contributed to the article and approved the final manuscript.

## Footnotes

1
http://surfer.nmr.mgh.harvard.edu/

2
http://www.rfmri.org/dpabi

3
http://www.fil.ion.ucl.ac.uk/spm/

4
https://www.mathworks.com/products/matlab.html
